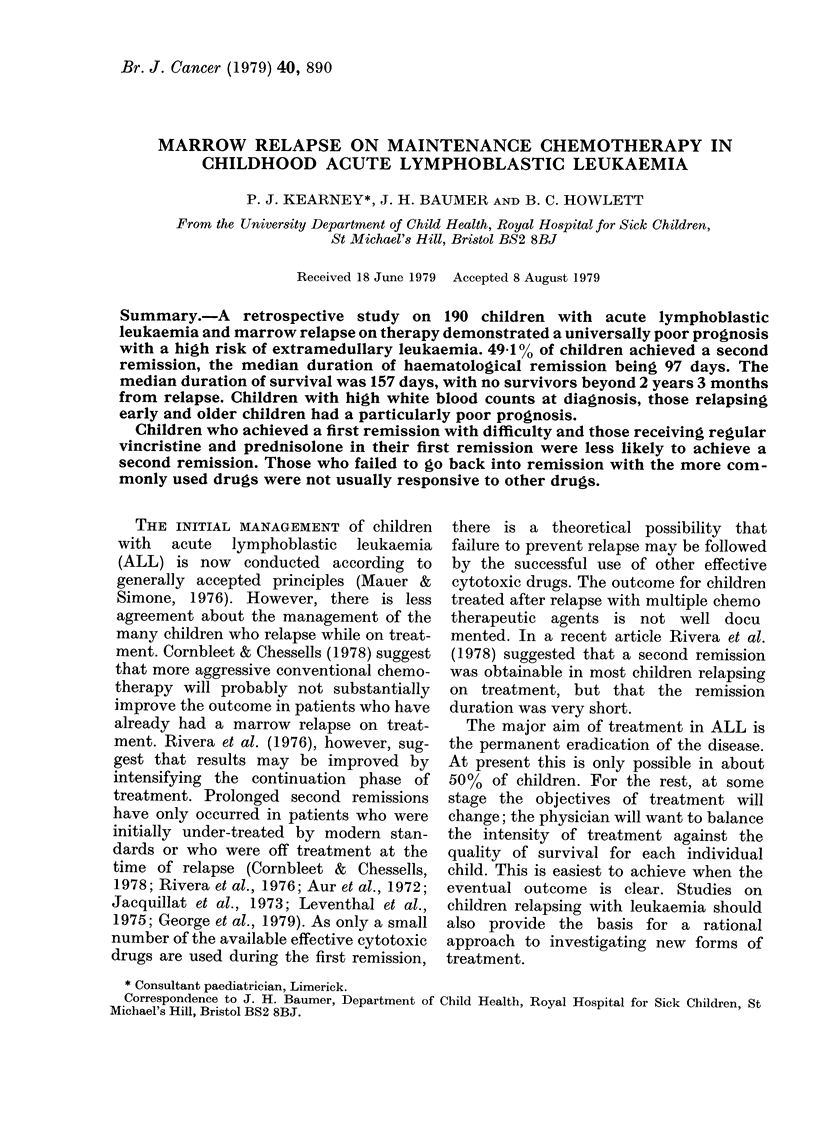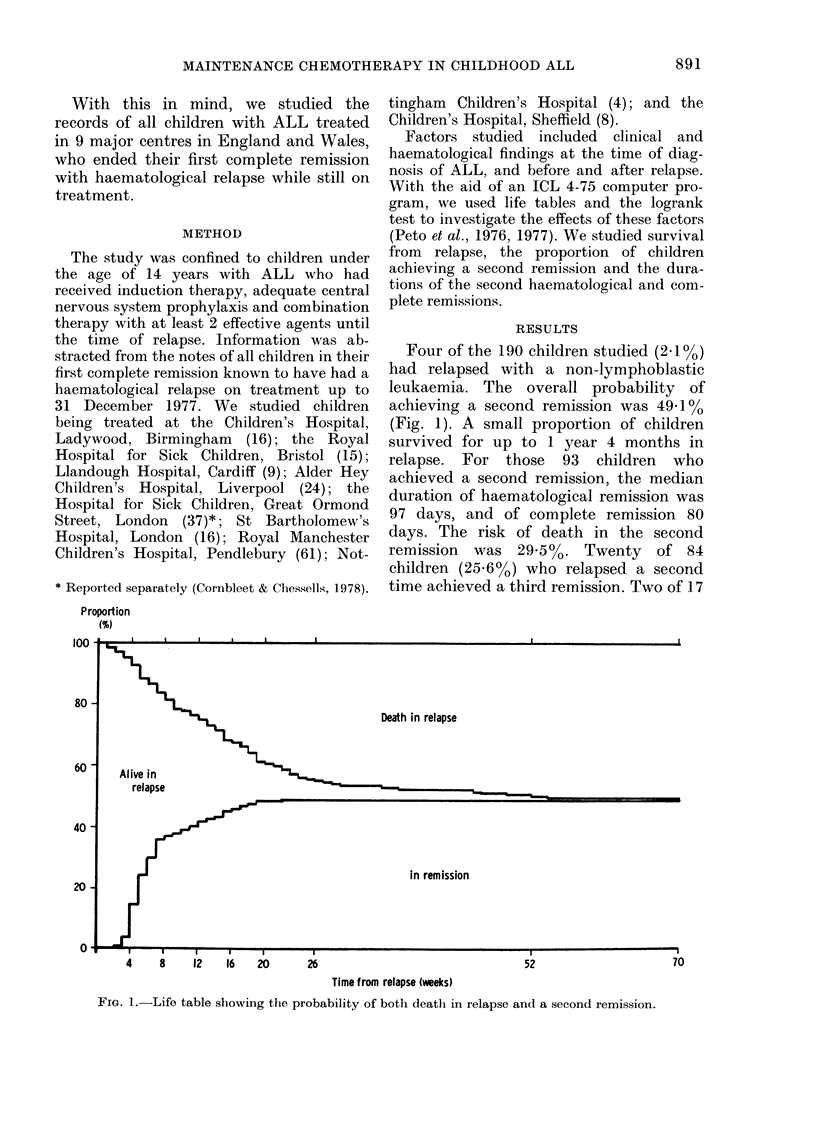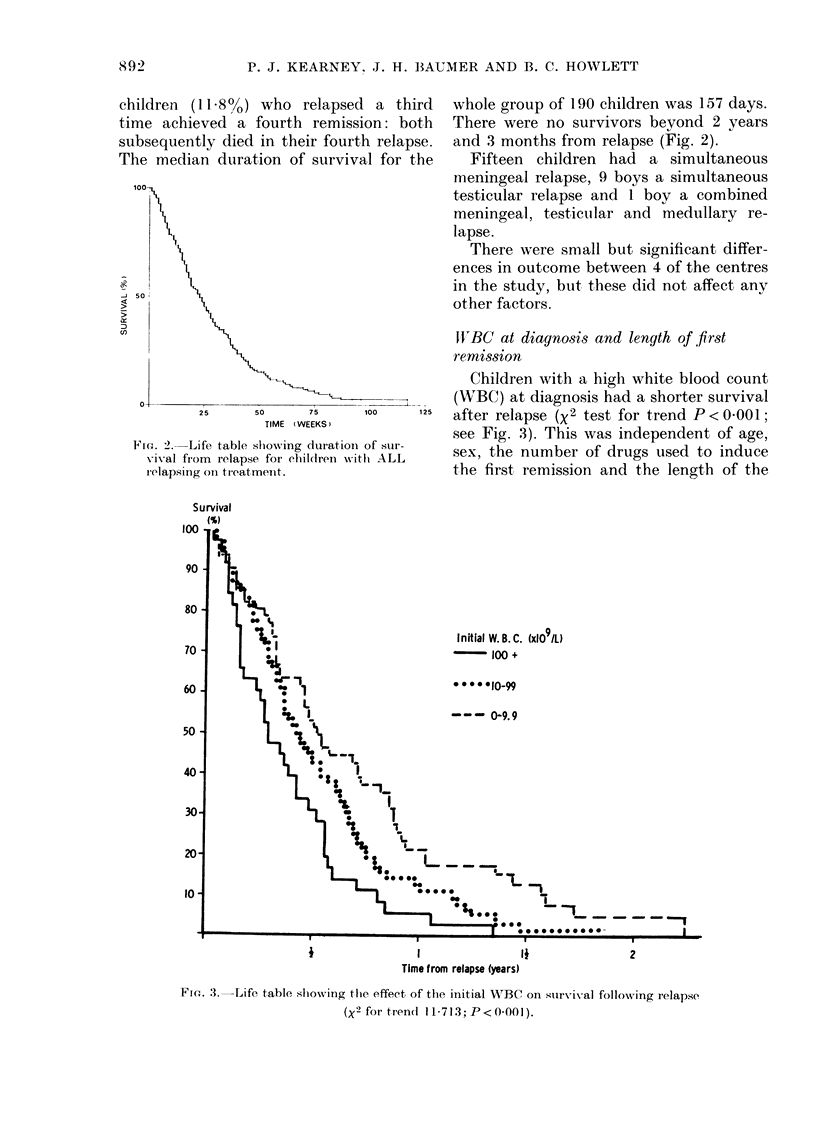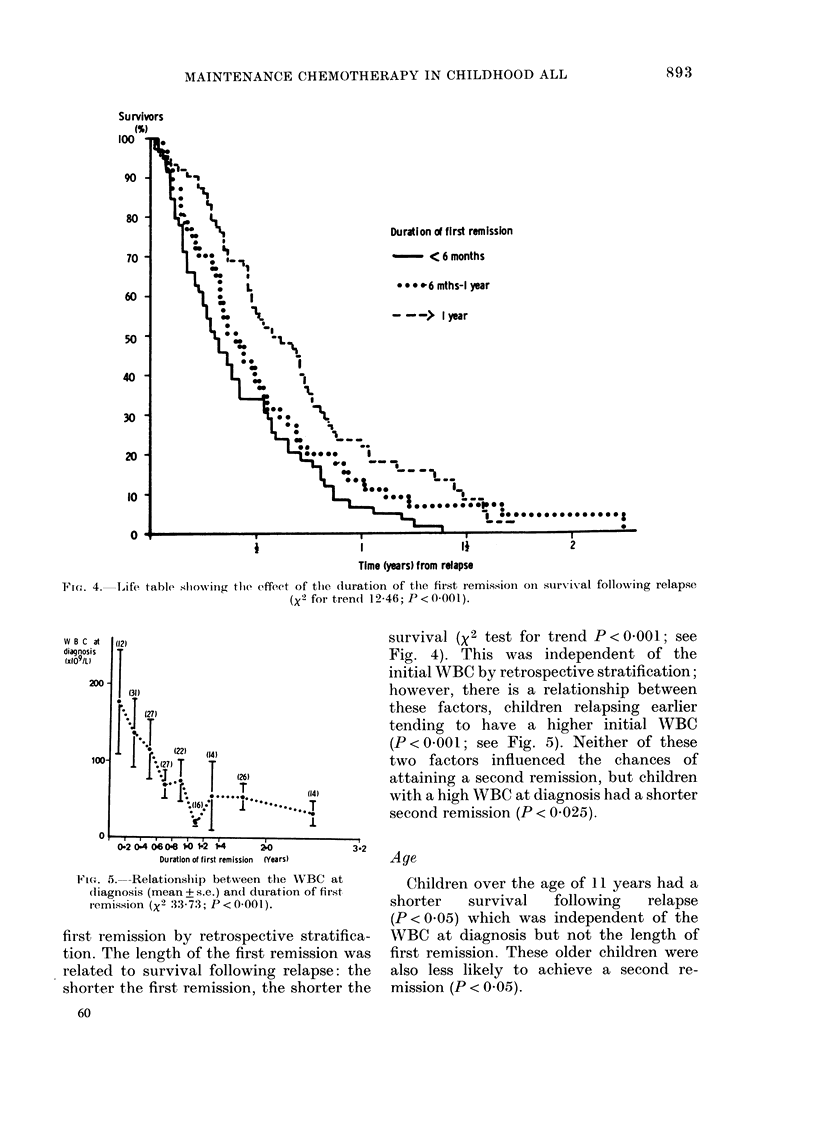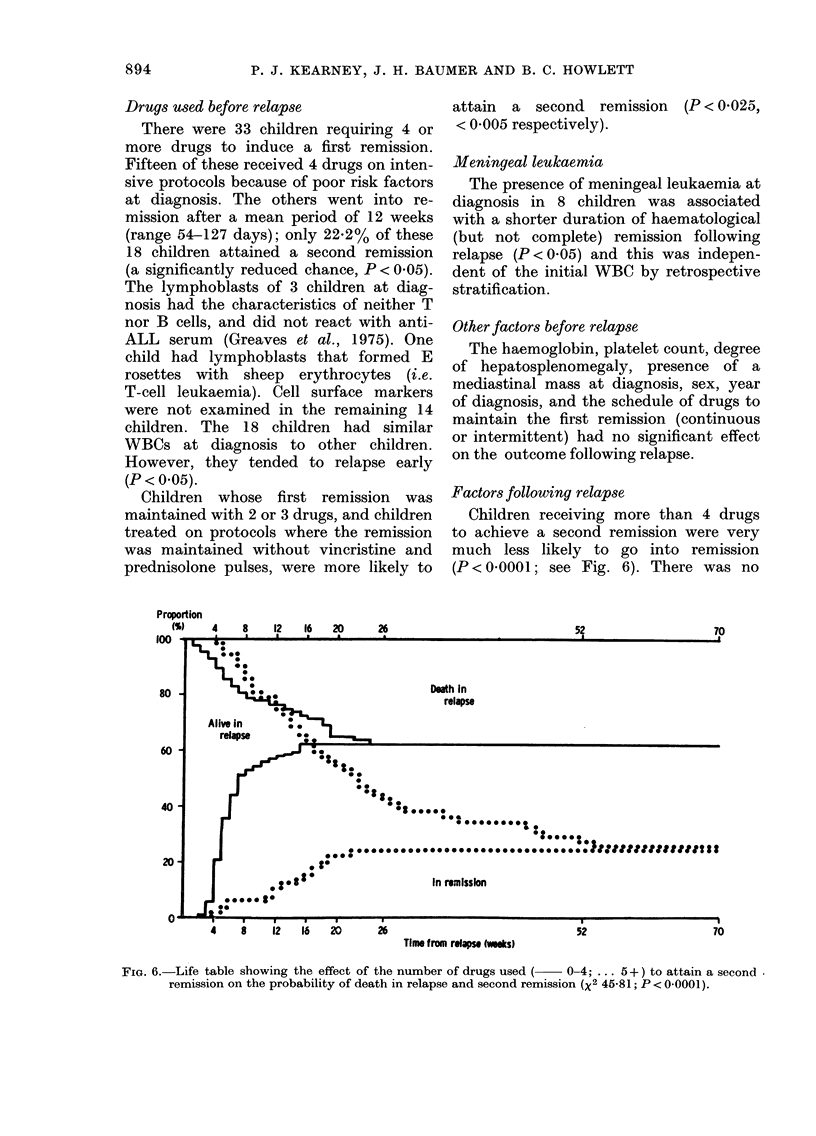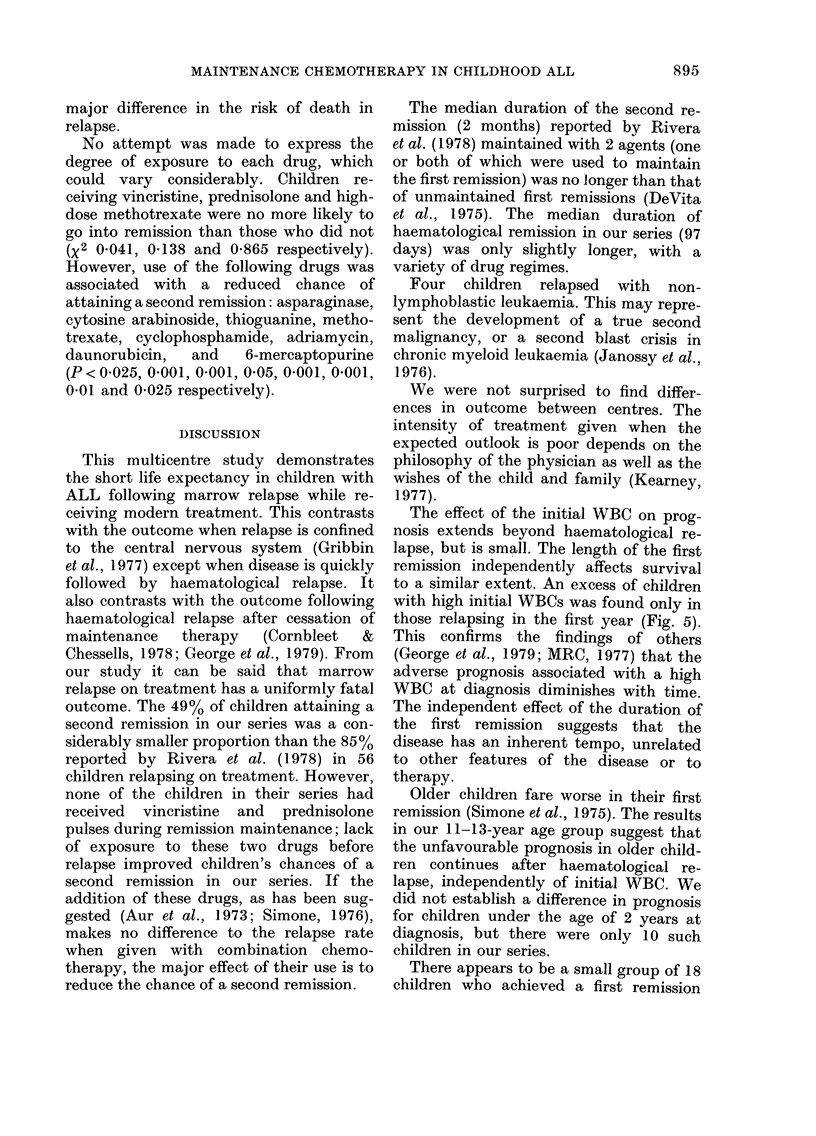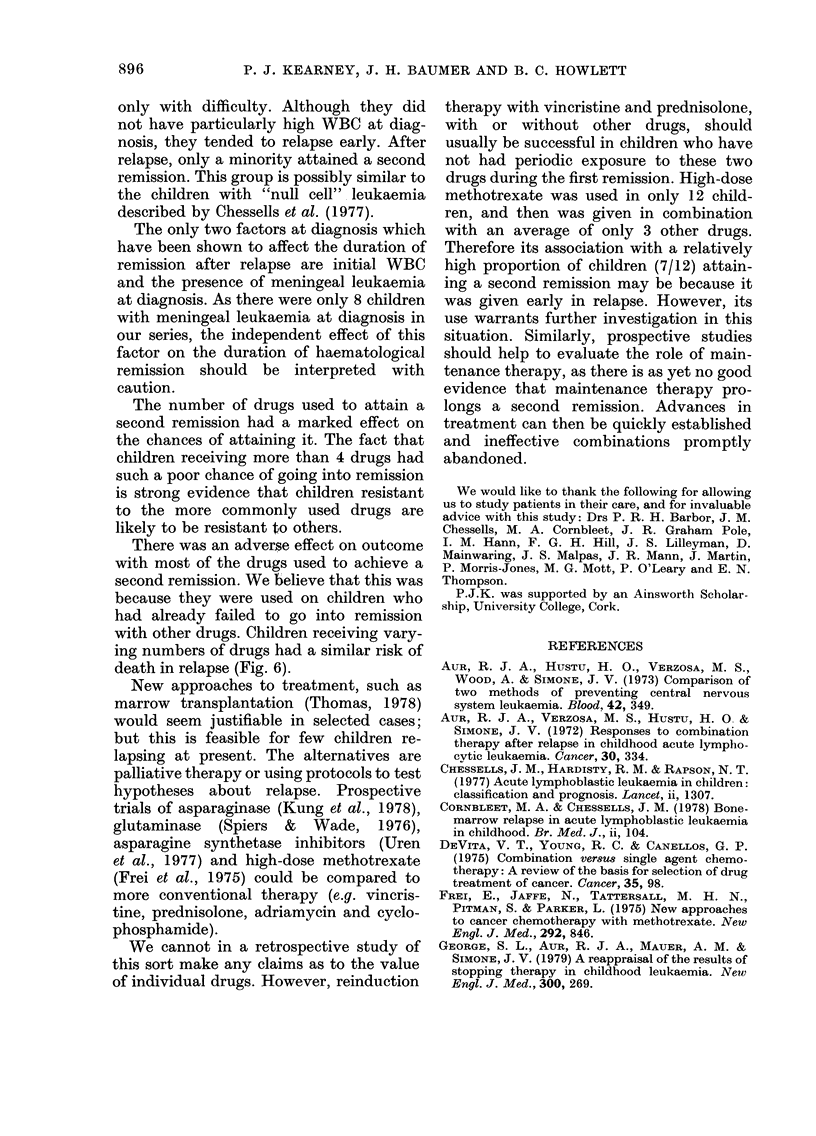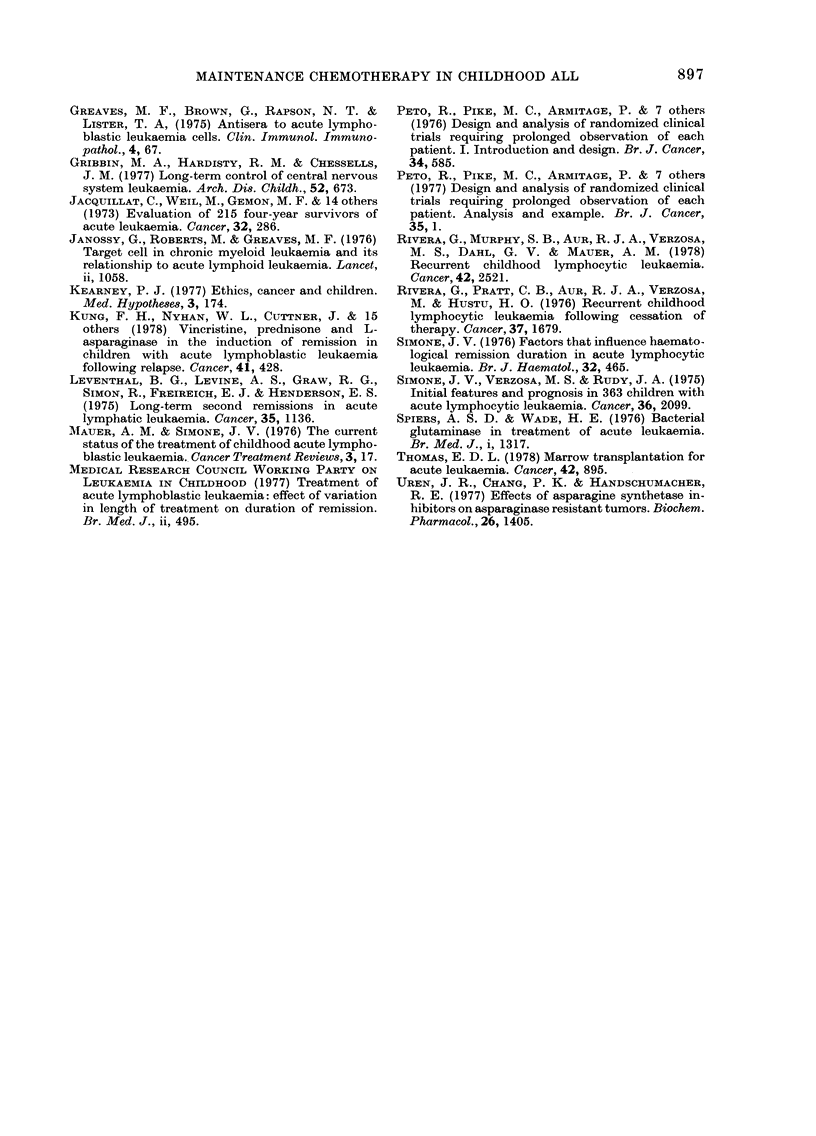# Marrow relapse on maintenance chemotherapy in childhood acute lymphoblastic leukaemia.

**DOI:** 10.1038/bjc.1979.282

**Published:** 1979-12

**Authors:** P. J. Kearney, J. H. Baumer, B. C. Howlett

## Abstract

A retrospective study on 190 children with acute lymphoblastic leukaemia and marrow relapse on therapy demonstrated a universally poor prognosis with a high risk of extramedullary leukaemia. 49.1% of children achieved a second remission, the median duration of haematological remission being 97 days. The median duration of survival was 157 days, with no survivors beyond 2 years 3 months from relapse. Children with high white blood counts at diagnosis, those relapsing early and older children had a particularly poor prognosis. Children who achieved a first remission with difficulty and those receiving regular vincristine and prednisolone in their remission were less likely to achieve a second remission. Those who failed to go back into remission with the more commonly used drugs were not usually responsive to other drugs.


*Br. J. Cancer* (1979) **40**, 890

### MARROW RELAPSE ON MAINTENANCE CHEMOTHERAPY IN CHILDHOOD ACUTE LYMPHOBLASTIC LEUKAEMIA

P. J. KEARNEY*, J. H. BAUMER AND B. C. HOWLETT

*From the University Department of Child Health, Royal Hospital for Sick Children, St Michael's Hill, Bristol BS2 8BJ*

Received 18 June 1979 Accepted 8 August 1979

**Summary.—A retrospective study on 190 children with acute lymphoblastic leukaemia and marrow relapse on therapy demonstrated a universally poor prognosis with a high risk of extramedullary leukaemia. 49·1% of children achieved a second remission, the median duration of haematological remission being 97 days. The median duration of survival was 157 days, with no survivors beyond 2 years 3 months from relapse. Children with high white blood counts at diagnosis, those relapsing early and older children had a particularly poor prognosis.**

**Children who achieved a first remission with difficulty and those receiving regular vincristine and prednisolone in their first remission were less likely to achieve a second remission. Those who failed to go back into remission with the more commonly used drugs were not usually responsive to other drugs.**

THE INITIAL MANAGEMENT of children with acute lymphoblastic leukaemia (ALL) is now conducted according to generally accepted principles (Mauer & Simone, 1976). However, there is less agreement about the management of the many children who relapse while on treatment. Cornbleet & Chessells (1978) suggest that more aggressive conventional chemotherapy will probably not substantially improve the outcome in patients who have already had a marrow relapse on treatment. Rivera *et al.* (1976), however, suggest that results may be improved by intensifying the continuation phase of treatment. Prolonged second remissions have only occurred in patients who were initially under-treated by modern standards or who were off treatment at the time of relapse (Cornbleet & Chessells, 1978; Rivera *et al.*, 1976; Aur *et al.*, 1972; Jacquillat *et al.*, 1973; Leventhal *et al.*, 1975; George *et al.*, 1979). As only a small number of the available effective cytotoxic drugs are used during the first remission, there is a theoretical possibility that failure to prevent relapse may be followed by the successful use of other effective cytotoxic drugs. The outcome for children treated after relapse with multiple chemo therapeutic agents is not well docu mented. In a recent article Rivera *et al.* (1978) suggested that a second remission was obtainable in most children relapsing on treatment, but that the remission duration was very short.

The major aim of treatment in ALL is the permanent eradication of the disease. At present this is only possible in about 50% of children. For the rest, at some stage the objectives of treatment will change; the physician will want to balance the intensity of treatment against the quality of survival for each individual child. This is easiest to achieve when the eventual outcome is clear. Studies on children relapsing with leukaemia should also provide the basis for a rational approach to investigating new forms of treatment.

* Consultant paediatrician, Limerick.

Correspondence to J. H. Baumer, Department of Child Health, Royal Hospital for Sick Children, St Michael's Hill, Bristol BS2 8BJ.

With this in mind, we studied the records of all children with ALL treated in 9 major centres in England and Wales, who ended their first complete remission with haematological relapse while still on treatment.

METHOD

The study was confined to children under the age of 14 years with ALL who had received induction therapy, adequate central nervous system prophylaxis and combination therapy with at least 2 effective agents until the time of relapse. Information was abstracted from the notes of all children in their first complete remission known to have had a haematological relapse on treatment up to 31 December 1977. We studied children being treated at the Children's Hospital, Ladywood, Birmingham (16); the Royal Hospital for Sick Children, Bristol (15); Llandough Hospital, Cardiff (9); Alder Hey Children's Hospital, Liverpool (24); the Hospital for Sick Children, Great Ormond Street, London (37)*; St Bartholomew's Hospital, London (16); Royal Manchester Children's Hospital, Pendlebury (61); Nottingham Children's Hospital (4); and the Children's Hospital, Sheffield (8).

* Reported separately (Cornbleet & Chessells, 1978).

Factors studied included clinical and haematological findings at the time of diagnosis of ALL, and before and after relapse. With the aid of an ICL 4-75 computer program, we used life tables and the logrank test to investigate the effects of these factors (Peto *et al.*, 1976, 1977). We studied survival from relapse, the proportion of children achieving a second remission and the durations of the second haematological and complete remissions.

RESULTS

Four of the 190 children studied (2·1%) had relapsed with a non-lymphoblastic leukaemia. The overall probability of achieving a second remission was 49·1% (Fig. 1). A small proportion of children survived for up to 1 year 4 months in relapse. For those 93 children who achieved a second remission, the median duration of haematological remission was 97 days, and of complete remission 80 days. The risk of death in the second remission was 29·5%. Twenty of 84 children (25·6%) who relapsed a second time achieved a third remission. Two of 17

FIG. 1.—Life table showing the probability of both death in relapse and a second remission.

children (11·8%) who relapsed a third time achieved a fourth remission: both subsequently died in their fourth relapse. The median duration of survival for the whole group of 190 children was 157 days. There were no survivors beyond 2 years and 3 months from relapse (Fig. 2).

FIG. 2.—Life table showing duration of survival from relapse for children with ALL relapsing on treatment.

Fifteen children had a simultaneous meningeal relapse, 9 boys a simultaneous testicular relapse and 1 boy a combined meningeal, testicular and medullary relapse.

There were small but significant differences in outcome between 4 of the centres in the study, but these did not affect any other factors.

### *WBC at diagnosis and length of first remission*

Children with a high white blood count (WBC) at diagnosis had a shorter survival after relapse ($\chi^2$ test for trend $P < 0{\cdot}001$; see Fig. 3). This was independent of age, sex, the number of drugs used to induce the first remission and the length of the

FIG. 3.—Life table showing the effect of the initial WBC on survival following relapse ($\chi^2$ for trend 11·713; $P < 0{\cdot}001$).

FIG. 4.—Life table showing the effect of the duration of the first remission on survival following relapse ($\chi^2$ for trend 12·46; $P < 0{\cdot}001$).

FIG. 5.—Relationship between the WBC at diagnosis (mean ± s.e.) and duration of first remission ($\chi^2$ 33·73; $P < 0{\cdot}001$).

first remission by retrospective stratification. The length of the first remission was related to survival following relapse: the shorter the first remission, the shorter the survival ($\chi^2$ test for trend $P < 0{\cdot}001$; see Fig. 4). This was independent of the initial WBC by retrospective stratification; however, there is a relationship between these factors, children relapsing earlier tending to have a higher initial WBC ($P < 0{\cdot}001$; see Fig. 5). Neither of these two factors influenced the chances of attaining a second remission, but children with a high WBC at diagnosis had a shorter second remission ($P < 0{\cdot}025$).

### *Age*

Children over the age of 11 years had a shorter survival following relapse ($P < 0{\cdot}05$) which was independent of the WBC at diagnosis but not the length of first remission. These older children were also less likely to achieve a second remission ($P < 0{\cdot}05$).

*Drugs used before relapse*

There were 33 children requiring 4 or more drugs to induce a first remission. Fifteen of these received 4 drugs on intensive protocols because of poor risk factors at diagnosis. The others went into remission after a mean period of 12 weeks (range 54–127 days); only 22·2% of these 18 children attained a second remission (a significantly reduced chance, $P < 0{\cdot}05$). The lymphoblasts of 3 children at diagnosis had the characteristics of neither T nor B cells, and did not react with anti-ALL serum (Greaves *et al.*, 1975). One child had lymphoblasts that formed E rosettes with sheep erythrocytes (*i.e.* T-cell leukaemia). Cell surface markers were not examined in the remaining 14 children. The 18 children had similar WBCs at diagnosis to other children. However, they tended to relapse early ($P < 0{\cdot}05$).

Children whose first remission was maintained with 2 or 3 drugs, and children treated on protocols where the remission was maintained without vincristine and prednisolone pulses, were more likely to attain a second remission ($P < 0{\cdot}025$, $< 0{\cdot}005$ respectively).

*Meningeal leukaemia*

The presence of meningeal leukaemia at diagnosis in 8 children was associated with a shorter duration of haematological (but not complete) remission following relapse ($P < 0{\cdot}05$) and this was independent of the initial WBC by retrospective stratification.

*Other factors before relapse*

The haemoglobin, platelet count, degree of hepatosplenomegaly, presence of a mediastinal mass at diagnosis, sex, year of diagnosis, and the schedule of drugs to maintain the first remission (continuous or intermittent) had no significant effect on the outcome following relapse.

*Factors following relapse*

Children receiving more than 4 drugs to achieve a second remission were very much less likely to go into remission ($P < 0{\cdot}0001$; see Fig. 6). There was no

FIG. 6.—Life table showing the effect of the number of drugs used (—— 0–4; . . . 5+) to attain a second remission on the probability of death in relapse and second remission ($\chi^2$ 45·81; $P < 0{\cdot}0001$).

major difference in the risk of death in relapse.

No attempt was made to express the degree of exposure to each drug, which could vary considerably. Children receiving vincristine, prednisolone and high-dose methotrexate were no more likely to go into remission than those who did not ($\chi^2$ 0·041, 0·138 and 0·865 respectively). However, use of the following drugs was associated with a reduced chance of attaining a second remission: asparaginase, cytosine arabinoside, thioguanine, methotrexate, cyclophosphamide, adriamycin, daunorubicin, and 6-mercaptopurine ($P < 0{\cdot}025$, 0·001, 0·001, 0·05, 0·001, 0·001, 0·01 and 0·025 respectively).

### DISCUSSION

This multicentre study demonstrates the short life expectancy in children with ALL following marrow relapse while receiving modern treatment. This contrasts with the outcome when relapse is confined to the central nervous system (Gribbin *et al.*, 1977) except when disease is quickly followed by haematological relapse. It also contrasts with the outcome following haematological relapse after cessation of maintenance therapy (Cornbleet & Chessells, 1978; George *et al.*, 1979). From our study it can be said that marrow relapse on treatment has a uniformly fatal outcome. The 49% of children attaining a second remission in our series was a considerably smaller proportion than the 85% reported by Rivera *et al.* (1978) in 56 children relapsing on treatment. However, none of the children in their series had received vincristine and prednisolone pulses during remission maintenance; lack of exposure to these two drugs before relapse improved children's chances of a second remission in our series. If the addition of these drugs, as has been suggested (Aur *et al.*, 1973; Simone, 1976), makes no difference to the relapse rate when given with combination chemotherapy, the major effect of their use is to reduce the chance of a second remission.

The median duration of the second remission (2 months) reported by Rivera *et al.* (1978) maintained with 2 agents (one or both of which were used to maintain the first remission) was no longer than that of unmaintained first remissions (DeVita *et al.*, 1975). The median duration of haematological remission in our series (97 days) was only slightly longer, with a variety of drug regimes.

Four children relapsed with non-lymphoblastic leukaemia. This may represent the development of a true second malignancy, or a second blast crisis in chronic myeloid leukaemia (Janossy *et al.*, 1976).

We were not surprised to find differences in outcome between centres. The intensity of treatment given when the expected outlook is poor depends on the philosophy of the physician as well as the wishes of the child and family (Kearney, 1977).

The effect of the initial WBC on prognosis extends beyond haematological relapse, but is small. The length of the first remission independently affects survival to a similar extent. An excess of children with high initial WBCs was found only in those relapsing in the first year (Fig. 5). This confirms the findings of others (George *et al.*, 1979; MRC, 1977) that the adverse prognosis associated with a high WBC at diagnosis diminishes with time. The independent effect of the duration of the first remission suggests that the disease has an inherent tempo, unrelated to other features of the disease or to therapy.

Older children fare worse in their first remission (Simone *et al.*, 1975). The results in our 11–13-year age group suggest that the unfavourable prognosis in older children continues after haematological relapse, independently of initial WBC. We did not establish a difference in prognosis for children under the age of 2 years at diagnosis, but there were only 10 such children in our series.

There appears to be a small group of 18 children who achieved a first remission only with difficulty. Although they did not have particularly high WBC at diagnosis, they tended to relapse early. After relapse, only a minority attained a second remission. This group is possibly similar to the children with "null cell" leukaemia described by Chessells *et al.* (1977).

The only two factors at diagnosis which have been shown to affect the duration of remission after relapse are initial WBC and the presence of meningeal leukaemia at diagnosis. As there were only 8 children with meningeal leukaemia at diagnosis in our series, the independent effect of this factor on the duration of haematological remission should be interpreted with caution.

The number of drugs used to attain a second remission had a marked effect on the chances of attaining it. The fact that children receiving more than 4 drugs had such a poor chance of going into remission is strong evidence that children resistant to the more commonly used drugs are likely to be resistant to others.

There was an adverse effect on outcome with most of the drugs used to achieve a second remission. We believe that this was because they were used on children who had already failed to go into remission with other drugs. Children receiving varying numbers of drugs had a similar risk of death in relapse (Fig. 6).

New approaches to treatment, such as marrow transplantation (Thomas, 1978) would seem justifiable in selected cases; but this is feasible for few children relapsing at present. The alternatives are palliative therapy or using protocols to test hypotheses about relapse. Prospective trials of asparaginase (Kung *et al.*, 1978), glutaminase (Spiers & Wade, 1976), asparagine synthetase inhibitors (Uren *et al.*, 1977) and high-dose methotrexate (Frei *et al.*, 1975) could be compared to more conventional therapy (*e.g.* vincristine, prednisolone, adriamycin and cyclophosphamide).

We cannot in a retrospective study of this sort make any claims as to the value of individual drugs. However, reinduction therapy with vincristine and prednisolone, with or without other drugs, should usually be successful in children who have not had periodic exposure to these two drugs during the first remission. High-dose methotrexate was used in only 12 children, and then was given in combination with an average of only 3 other drugs. Therefore its association with a relatively high proportion of children (7/12) attaining a second remission may be because it was given early in relapse. However, its use warrants further investigation in this situation. Similarly, prospective studies should help to evaluate the role of maintenance therapy, as there is as yet no good evidence that maintenance therapy prolongs a second remission. Advances in treatment can then be quickly established and ineffective combinations promptly abandoned.

We would like to thank the following for allowing us to study patients in their care, and for invaluable advice with this study: Drs P. R. H. Barbor, J. M. Chessells, M. A. Cornbleet, J. R. Graham Pole, I. M. Hann, F. G. H. Hill, J. S. Lilleyman, D. Mainwaring, J. S. Malpas, J. R. Mann, J. Martin, P. Morris-Jones, M. G. Mott, P. O'Leary and E. N. Thompson.

P.J.K. was supported by an Ainsworth Scholarship, University College, Cork.

### REFERENCES

AUR, R. J. A., HUSTU, H. O., VERZOSA, M. S., WOOD, A. & SIMONE, J. V. (1973) Comparison of two methods of preventing central nervous system leukaemia. *Blood*, **42**, 349.

AUR, R. J. A., VERZOSA, M. S., HUSTU, H. O. & SIMONE, J. V. (1972) Responses to combination therapy after relapse in childhood acute lymphocytic leukaemia. *Cancer*, **30**, 334.

CHESSELLS, J. M., HARDISTY, R. M. & RAPSON, N. T. (1977) Acute lymphoblastic leukaemia in children: classification and prognosis. *Lancet*, ii, 1307.

CORNBLEET, M. A. & CHESSELLS, J. M. (1978) Bone-marrow relapse in acute lymphoblastic leukaemia in childhood. *Br. Med. J.*, ii, 104.

DEVITA, V. T., YOUNG, R. C. & CANELLOS, G. P. (1975) Combination *versus* single agent chemotherapy: A review of the basis for selection of drug treatment of cancer. *Cancer*, **35**, 98.

FREI, E., JAFFE, N., TATTERSALL, M. H. N., PITMAN, S. & PARKER, L. (1975) New approaches to cancer chemotherapy with methotrexate. *New Engl. J. Med.*, **292**, 846.

GEORGE, S. L., AUR, R. J. A., MAUER, A. M. & SIMONE, J. V. (1979) A reappraisal of the results of stopping therapy in childhood leukaemia. *New Engl. J. Med.*, **300**, 269.

GREAVES, M. F., BROWN, G., RAPSON, N. T. & LISTER, T. A, (1975) Antisera to acute lymphoblastic leukaemia cells. *Clin. Immunol. Immunopathol.*, **4**, 67.

GRIBBIN, M. A., HARDISTY, R. M. & CHESSELLS, J. M. (1977) Long-term control of central nervous system leukaemia. *Arch. Dis. Childh.*, **52**, 673.

JACQUILLAT, C., WEIL, M., GEMON, M. F. & 14 others (1973) Evaluation of 215 four-year survivors of acute leukaemia. *Cancer*, **32**, 286.

JANOSSY, G., ROBERTS, M. & GREAVES, M. F. (1976) Target cell in chronic myeloid leukaemia and its relationship to acute lymphoid leukaemia. *Lancet*, ii, 1058.

KEARNEY, P. J. (1977) Ethics, cancer and children. *Med. Hypotheses*, **3**, 174.

KUNG, F. H., NYHAN, W. L., CUTTNER, J. & 15 others (1978) Vincristine, prednisone and L-asparaginase in the induction of remission in children with acute lymphoblastic leukaemia following relapse. *Cancer*, **41**, 428.

LEVENTHAL, B. G., LEVINE, A. S., GRAW, R. G., SIMON, R., FREIREICH, E. J. & HENDERSON, E. S. (1975) Long-term second remissions in acute lymphatic leukaemia. *Cancer*, **35**, 1136.

MAUER, A. M. & SIMONE, J. V. (1976) The current status of the treatment of childhood acute lymphoblastic leukaemia. *Cancer Treatment Reviews*, **3**, 17.

MEDICAL RESEARCH COUNCIL WORKING PARTY ON LEUKAEMIA IN CHILDHOOD (1977) Treatment of acute lymphoblastic leukaemia: effect of variation in length of treatment on duration of remission. *Br. Med. J.*, ii, 495.

PETO, R., PIKE, M. C., ARMITAGE, P. & 7 others (1976) Design and analysis of randomized clinical trials requiring prolonged observation of each patient. I. Introduction and design. *Br. J. Cancer*, **34**, 585.

PETO, R., PIKE, M. C., ARMITAGE, P. & 7 others (1977) Design and analysis of randomized clinical trials requiring prolonged observation of each patient. Analysis and example. *Br. J. Cancer*, **35**, 1.

RIVERA, G., MURPHY, S. B., AUR, R. J. A., VERZOSA, M. S., DAHL, G. V. & MAUER, A. M. (1978) Recurrent childhood lymphocytic leukaemia. *Cancer*, **42**, 2521.

RIVERA, G., PRATT, C. B., AUR, R. J. A., VERZOSA, M. & HUSTU, H. O. (1976) Recurrent childhood lymphocytic leukaemia following cessation of therapy. *Cancer*, **37**, 1679.

SIMONE, J. V. (1976) Factors that influence haematological remission duration in acute lymphocytic leukaemia. *Br. J. Haematol.*, **32**, 465.

SIMONE, J. V., VERZOSA, M. S. & RUDY, J. A. (1975) Initial features and prognosis in 363 children with acute lymphocytic leukaemia. *Cancer*, **36**, 2099.

SPIERS, A. S. D. & WADE, H. E. (1976) Bacterial glutaminase in treatment of acute leukaemia. *Br. Med. J.*, i, 1317.

THOMAS, E. D. L. (1978) Marrow transplantation for acute leukaemia. *Cancer*, **42**, 895.

UREN, J. R., CHANG, P. K. & HANDSCHUMACHER, R. E. (1977) Effects of asparagine synthetase inhibitors on asparaginase resistant tumors. *Biochem. Pharmacol.*, **26**, 1405.